# THE IMPACT OF LIVING IN MULTIGENERATIONAL NEIGHBOURHOODS ON LONELINESS IN LATER LIFE

**DOI:** 10.1093/geroni/igad104.1931

**Published:** 2023-12-21

**Authors:** Martin Hyde, Elizabeth Evans

**Affiliations:** Swansea University, Swansea, Wales, United Kingdom; Swansea University, Swansea, Wales, United Kingdom

## Abstract

Intergenerational solidarity has been identified as a key factor for effective social functioning. However, evidence suggests that divisions between younger and older generations within Great Britain (GB) have never been greater. Growing residential age segregation is thought to be driving intergenerational division. This, in turn, is assumed to lead to an increased risk of loneliness among older adults. However, to date, this has not been empirically tested. To examine this issue, we created a new measure of multigenerational neighbourhoods, for all lower super output areas in England and Wales. These were linked to individuals aged 50 and over living in England and Wales in 2018/2019 in the UK Household Longitudinal Survey (N = 14,196). Loneliness was measured using the UCLA-3 scale. Multilevel regression analyses were performed. Covariates included, area level deprivation, urban/rural, residential tax band, home ownership, individual age, sex, marital status and financial situation. The results show that the mix of generations within a neighbourhood has a weak (r = 0.33) and non-significant (p=.493) association with loneliness among older adults. To the best of our knowledge this is the first study to directly examine the impact of neighbourhood age diversity on loneliness in later life. Our findings suggest that the mere presence of different generations within a neighbourhood is not a sufficient condition to combat loneliness in later life

